# Gene expression profiles identify key factors in inflammatory odontoblastic dental pulp stem cell differentiation via TNFα/C5L2

**DOI:** 10.3389/fgene.2025.1592599

**Published:** 2025-06-04

**Authors:** Muhammad Irfan, Ji Hyun Kim, Sreelekshmi Sreekumar, Seung Chung

**Affiliations:** Department of Oral Biology, College of Dentistry, University of Illinois Chicago, Chicago, IL, United States

**Keywords:** RNA sequencing, DPSCs, TNFα, inflammation, dentinogenesis, regeneration TNFα stimulates DPSCs odontoblastic differentiation

## Abstract

**Introduction:**

Inflammation is a complex host response to harmful infections or injuries, playing beneficial and detrimental roles in tissue regeneration. Notably, clinical dentinogenesis associated with caries development occurs within an inflammatory environment. Reparative dentinogenesis is closely linked to intense inflammation, which triggers the recruitment and differentiation of dental pulp stem cells (DPSCs) into the dentin lineage. Understanding how inflammatory responses influence DPSCs is essential for elucidating the mechanisms underlying dentin and pulp regeneration.

**Methods:**

Given the limited data on this process, a broad approach is employed here to understand better the complex mechanisms involved and identify downstream signaling targets. This study investigates the role of inflammation and the complement receptor C5L2 in the odontoblastic differentiation of DPSCs and the associated transcriptomic changes using poly-A RNA sequencing (RNA-seq). RNA-seq techniques provide insight into the transcriptome of a cell, offering higher coverage and greater resolution of its dynamic nature.

**Results:**

Following inflammatory stimulation, DPSCs exhibit significantly altered gene profiles, including marked up-regulation of key odontogenic genes, highlighting the critical role of inflammation in dentinogenesis. We demonstrate that TNFα-treated, odontoblast-like differentiating DPSCs, under C5L2 modulation, show differentially expressed gene profiles and transcriptomic changes.

**Conclusion:**

Beyond quantifying gene expression, RNA-seq data also enable the discovery of novel transcripts, the identification of alternatively spliced genes, and the detection of allele-specific expression. The data presented may offer new avenues for experimental approaches to uncovering pathways in dentinogenesis by identifying specific transcription factors and gene profiles.

## 1 Introduction

Dental caries is a common infectious pathology that affects the oral tissues, caused by the invasion of bacteria into the enamel and dentin (Mallya and Mallya, 2020). Current treatment options are limited and often lead to the irreversible loss of pulp tissue (Schwendicke et al., 2015). The advancement of bacterial caries into dentin prompts dental pulp stem cells (DPSCs) to migrate and differentiate at the site of infection, initiating a regenerative process that leads to the formation of “tertiary” dentin (Fawzy El-Sayed et al., 2019). This odontoblastic differentiation of DPSCs in response to caries occurs within an inflammatory environment. Inflammation is a crucial immune response that plays a key role in protecting the body from infection and tissue damage. Its complex roles in tissue regeneration, both positive and negative, have gained increasing attention (Cooke, 2019). Recent studies have shown that inflammation is instrumental in the regeneration of damaged dental tissues by promoting the recruitment and proliferation of pulp progenitor cells and the differentiation of odontoblasts (Cooper et al., 2014). If this delicate balance between inflammation and regeneration is disrupted, it can lead to irreversible tissue damage. However, there is still limited understanding of the role of inflammation in reparative dentinogenesis and the underlying biology of DPSCs.

Inflammatory cells are recruited to an injury site and release cytokines and growth factors (Barrientos et al., 2008). In this study, we utilized the inflammatory cytokine Tumor Necrosis Factor-alpha (TNFα), which is known to be elevated in patients with dental caries (Pezelj-Ribaric et al., 2002) and plays a central role in coordinating various cellular signaling events (Qin et al., 2015; Shin et al., 2015). Notably, TNFα and ROS, at low levels, were reported to promote reparative dentinogenesis (Cooper et al., 2014). TNFα is one of the several pro-inflammatory cytokines involved in bone tissue remodeling and regulation of homeostasis by stimulating osteoclastogenesis and inhibiting osteoblast function. It is also involved in the pathogenesis of chronic inflammation and a few inflammatory diseases, such as ulcerative colitis and rheumatoid arthritis. However, findings suggest that it also induces osteogenic differentiation (Osta et al., 2014).

Complement C5a second receptor (C5aR2/C5L2), an alternate C5a receptor, plays a significant role in regeneration and inflammation (Mastellos et al., 2013). C5L2-deficient mice exhibited increased mortality, hepatic necrosis, and impaired liver regeneration following partial hepatectomy, emphasizing its critical function in these processes. Our previous study demonstrated that C5L2 modulates brain-derived neurotrophic factor (BDNF) secretion in human dental pulp stem cells (DPSCs). Silencing C5L2 resulted in increased BDNF production, which may accelerate nerve regeneration. This effect is mediated via the p38^MAPK^ pathway, as shown in our findings (Irfan and Chung, 2023). We recently established that inflammation is directly involved in complement C5a-mediated dentin repair (Irfan et al., 2024; Kim et al., 2024). Here, we ought to study the gene regulatory effects of TNFα stimulation with C5L2 silencing on DPSCs odontoblastic differentiation.

An alternative therapeutic approach involves regenerating dentin and pulp using biomimetic tissue and stem cell engineering. In regenerative medicine, stem cells have been proposed over the last decade, with attention focused on mesenchymal stem cells (MSCs) as a therapeutic option due to their protective and regenerative abilities (Vizoso et al., 2017). Growing evidence indicates the importance of paracrine signaling induced by MSCs as a supportive mechanism of regeneration of the respective damaged tissue (Kumar et al., 2019; Harrell et al., 2019; Luzuriaga et al., 2021). Currently, there is growing evidence that human DPSCs have many similarities to bone marrow-derived MSCs (BMMSCs) and can be easily isolated, thus having a clear advantage over the costly and invasive techniques required with MSCs collected from bone marrow and DPSCs can be isolated from various dental soft tissues without any invasive technique, such as human dental pulp tissue of permanent teeth (Tsutsui, 2020). Regarding stem cell therapy and regeneration capacity, several advantages and superiority of DPSCs over MSCs have been reported (Pagella et al., 2020; Irfan et al., 2022a). Not only stem cell markers, but DPSCs also express dentin and odontoblast differentiation markers such as dentin sialophosphoprotein (DSPP), dentin matrix protein-1 (DMP-1), alkaline phosphatases (ALP), and type I collagen (Anitua et al., 2018). DPSCs can be differentiated by modulation with growth factors, transcriptional factors, extracellular matrix proteins, tissues, and cells, including osteoblasts, odontoblasts, neuron cells, and hepatocytes (Gronthos et al., 2000). Taken together, these aspects represent DPSCs as promising sources in the dentin-pulp regeneration complex.

Previously, gene expression studies relied on low-throughput methods, such as northern blots and quantitative polymerase chain reaction (qPCR), limited to measuring sole transcripts. Methodologies have been advanced to enable genome-wide quantification of gene expression, better known as transcriptomics. A high-throughput next-generation sequencing has revolutionized transcriptomics by allowing RNA analysis and understanding of the complex and dynamic nature of the transcriptome (Wang et al., 2009). In this study, we have investigated the transitional aspects of inflammation, its effects, and underlying mechanisms by examining changes in differential gene profiles and downstream transcriptional factors. Specifically, we analyzed odontoblast-like differentiation of DPSCs treated with TNFα and with or without C5L2 silencing through poly-A RNA sequencing to identify key genes and transcription factors involved in the odontogenic differentiation process.

## 2 Materials and methods

### 2.1 Chemicals and reagents

Human DPSCs were purchased from Lonza, Pharma & Biotech (Cat. # PT-5025). MEM-alpha, DMEM, PBS, fetal bovine serum, L-glutamine, and Antibiotic–Antimycotic were procured from Gibco™ Fisher Scientific (Waltham, MA, United States). Poly-D-Lysine-coated (BioCoat™, 12 mm) round German glass coverslips were purchased from Corning™ Fisher Scientific (Cat. # 354087; Waltham, MA, United States). Human recombinant TNFα was from Invitrogen, Fisher Scientific (Waltham, MA, United States), RIPA buffer was from Cell Signaling (Danvers, MA, United States), and the DMP-1 ELISA kit was from Invitrogen, Thermo Fisher Scientific (Waltham, MA, United States), and a few other chemicals were from Fisher Chemical (Nazareth, PA, United States). Primer Sequences were purchased from IDT. siRNA targeting human C5L2, siRNA control, and siRNA Reagent System were purchased from Santa Cruz Biotechnology (Dallas, TX, United States).

### 2.2 Cell culture and treatments

Human DPSCs were commercially procured (guaranteed through 10 population doublings to express CD105, CD166, CD29, CD90, and CD73, and not to express CD34, CD45, and CD133), which were further evaluated by immunocytochemistry in cultures with STRO-1, a stem cell marker. DPSCs were cultured at 37°C and 5% CO_2_ for 3 days in regular growth media (αMEM containing 10% fetal bovine serum, 1% L-glutamine), and cells were transfected using the siRNA Reagent System as previously described (Irfan and Chung, 2023). Then, cells were differentiated in dentinogenic media (DMEM containing 20% FBS, 1% L-glutamine, and antimycotic/antibiotic, i.e., 100 μg/mL streptomycin, 100 U/mL penicillin, supplemented with 100 μg/mL ascorbic acid, 10 mmol/L β-glycerophosphate, and 10 mmol/L dexamethasone) for 1-week, treated with or without TNFα (10 ng/mL) every 3 days. All the experiments were conducted with different sets of DPSCs (between the 2nd and 4th passages) 3 times, and cell proliferation was measured by counting the total number of cells.

### 2.3 Silencing of C5L2 expression by siRNA

Human DPSCs were cultured in a 6-well plate in an antibiotic-free medium with up to 60%–80% confluence. Transient transfection with siRNAs was performed using the siRNA Reagent System (sc-45064) according to the manufacturer´s protocol as previously described (Irfan and Chung, 2023). Cells were incubated in an antibiotic and serum-free transfection solution containing a mixture of transfection reagent and 40 pmol/mL/well of C5L2 siRNA (sc-105165) or control siRNA, which is a non-targeting siRNA designed as a negative control (sc-37007). After 6 h, 1 mL of medium containing 2 times the normal serum and antibiotic concentration was added to each well without removing the transfection mixture. After 24 h, the medium was replaced with a fresh dentinogenic medium and incubated further accordingly, as mentioned above.

### 2.4 Total RNA-seq with GO and KEGG enrichment analyses

LC Sciences performed RNA-seq from RNA isolated from DPSCs pallets collected after 1 week of treatment with or without TNFα or siC5L2 between passages 2–4. RNA was isolated using the Qiagen RNeasy Mini Kit (79,216 Qiagen, Germantown, MD). Poly(A) RNA sequencing library was prepared following Illumina’s TruSeq-stranded-mRNA sample preparation protocol. The total RNA quality, quantity, and purity were analyzed by Bioanalyzer 2,100 and RNA 6,000 Nano LabChip Kit (Agilent, CA, United States, 5,067–1,511), and high-quality RNA samples with RIN number >7.0 were used to construct a sequencing library. Poly(A) tail-containing mRNAs were purified using oligo-(dT) magnetic beads with double rounds of purification. Then, it was fragmented into short fragments using a divalent cation buffer under higher temperatures (Magnesium RNA Fragmentation Module (NEB, cat. No. e6150, United States) under 94°C 5- 7 min). Paired-end sequencing was performed on Illumina’s NovaSeq 6,000 sequencing system. To remove the reads with adaptor contamination, low-quality bases, and undetermined bases, in-house scripts were used, and bioinformatics analysis was performed (Martin, 2011). FastQC (http://www.bioinformatics.babraham.ac.uk/projects/fastqc/) verified the sequence quality. HISAT2 (Kim et al., 2015) was used to map reads to the genome of http://ftp.ensembl.org/pub/release-101/fasta/mus_musculus/dna/. The mapped reads of each sample were assembled using StringTie (Pertea et al., 2015). Then, all transcriptomes were merged to reconstruct a comprehensive transcriptome using Perl scripts and gffcompare. After the generation of final transcriptome, StringTie (Pertea et al., 2015) and edgeR (Robinson et al., 2010) were used to assess the expression levels of all transcripts. StringTie (Pertea et al., 2015) was used to perform mRNA expression levels by calculating FPKM. The differential gene expression analysis was performed by Ballgown. The differentially expressed mRNAs were selected with log2 (fold change) > 1 or log2 (fold change) < −1 and with statistical significance (p-value < 0.05) by the R package edgeR (Robinson et al., 2010). Three biological replicates were used for the analysis.

### 2.5 Real-time PCR

Human DPSCs were cultured in a 6-well plate at 5 × 10^4^ cells/well for up to 1 week in dentinogenic media. RNeasy Mini Kit (Qiagen, Hilden, Germany) was used to extract and analyze mRNA using the Fisher Scientific NanoDrop 2000 device. According to the manufacturer’s protocol, the cDNA samples were analyzed using the Applied Biosystems SYBR green reagent system. The target gene expressions were standardized by the housekeeping gene GAPDH using the 2^−ΔΔCT^ method (Livak and Schmittgen, 2001).

### 2.6 Alizarin red staining

The six-well plates were washed two times with distilled water and fixed with 4% PFA for 1 h at RT. The cells were then washed with distilled water two times, and 1 mL of 40 mM of alizarin red stain (ARS) was added per well; the ARS was provided by ScienCell (#8678). After 1 h of gentle shaking, the plate was washed with distilled water (pH 4) three times and dried. The cells were inspected using a Leica DMi1 phase microscope, and images were obtained. Then, 10% acetic acid solution was added to the wells for 30 min at RT with gentle shaking, and samples were collected and processed with 10% ammonium hydroxide for ARS quantification. The plot was drawn against standards and respective samples.

### 2.7 DMP-1 quantitative ELISA

Supernatants from DPSC culture, incubated with various abovementioned treatments, were collected from cultures after 7 days of differentiation and assayed using the ELISA kit according to the manufacturer’s protocol. Then, a standard curve was constructed using standards and test samples in duplicate at increasing concentrations, and values were normalized accordingly.

### 2.8 Statistical analysis

The statistical analyses were performed on at least 3 independent experiments with duplicates or triplicates, and statistical significance was determined using one-way analysis of variance (ANOVA) followed by posthoc Dunnett’s test (SAS 9.4) to compare the different treatments and their respective controls (p-value of 0.05 or less was considered statistically significant). In addition, the data were also analyzed by Tukey’s test for statistical significance between the groups.

## 3 Results

### 3.1 Functional validation of odontoblast-like differentiating DPSCs and their mineralization activity

**FIGURE 1:**
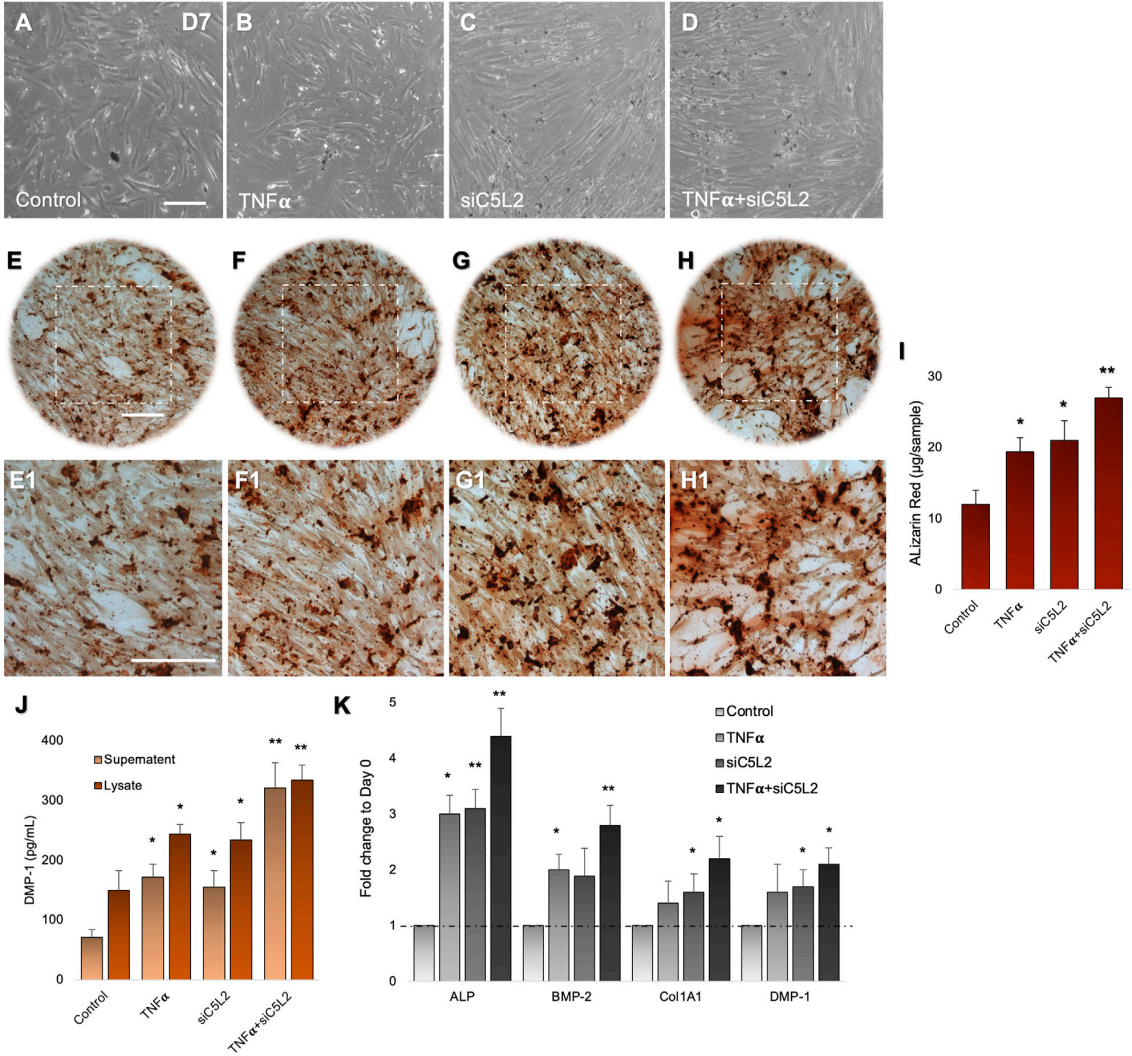
Mineralization activity of differentiated DPSCs in dentinogenic media and their functional validation of odontoblast-like effects. **(A–D)** Differentiated odontoblast-like DPSCs in dentinogenic media at day 7 under various conditions. **(E–H1)** Mineralization activity of differentiated DPSCs in dentinogenic media. Cells were stained with ARS, and images were taken using a Leica DMi1 phase microscope. Images show higher mineralization matrix in the TNFα alone **(F,F1)** or siC5L2 **(G,G1)** or their combined treated group **(H,H1)** compared with the control **(E,E1)**. Scale bars: 100 μm. **(I)** Bar graph shows ARS quantification among various treatment groups indicating significantly higher peaks in the siC5L2, TNFα, or their combined treatment. **(J)** Effects of siC5L2 or TNFα on the secretion of DMP-1 in supernatants and lysates of odontoblast-like differentiated DPSCs. Cells were cultured and differentiated under various conditions. The supernatants (conditioned media) and lysates were collected at day 7, and an ELISA was performed to quantify DMP-1 secretion according to the manufacturer’s protocol. DMP-1 production was significantly increased in the siC5L2 cells with or without TNFα. **(K)** The expression of odontoblastic markers ALP, BMP-2, COL1A1, and DMP-1 mRNA during the odontogenic differentiation was quantified by real-time PCR. The elevated level of these markers represents the odontoblast-like differentiation of DPSCs. The graph bars show the mean ± SD of at least three independent experiments (n = 3) in duplicate. *p < 0.05, **p < 0.01, and ***p < 0.001 vs. control.

DPSCs were differentiated in the dentinogenic medium for 7 days after C5L2 silencing, with or without TNFα stimulation. Under a light microscope observation, differentiated cells showed mineralization, analyzed by experts blinded to the study, which was further confirmed through various other experiments. siC5L2 and TNFα groups showed more mineralization (Figures 1B–D) compared to the control differentiated group (Figure 1A). Then, differentiated cells were stained with ARS to analyze the formation of a mineralization matrix surrounding the microenvironment during dentinogenic differentiation. The siC5L2 group (Figures 1F,F1) and TNFα group (Figures 1G,G1) show more mineralization matrix than the control (Figures 1E,E1), while their combined effect drastically enhanced mineralization phenotypes (Figures 1H,H1). Graph bars show significantly higher mineralization in siC5L2 groups with or without TNFα treatment (Figure 1I, p < 0.05, p < 0.01).

To complement mineralization data, we performed ELISA. Our data show C5L2-mediated DMP-1 regulations in odontoblast-like differentiating DPSCs (Figure 1J). The co-treated group showed the most significant production of DMP-1 in the TNFα and siC5L2 (p < 0.01) groups compared with the alone (p < 0.05) or control groups. These results suggest that the siC5L2-treated group secretes higher levels of mineralization markers, and TNFα stimulation enhances the effect of C5L2 silencing. To further validate the proposed *in vitro* model for studying odontoblast-like cell differentiation from DPSCs, alkaline phosphatase (ALP), bone morphogenic proteins 2 (BMP-2), type 1 collagen (Col1A1), and DMP-1 were analyzed using real-time PCR, and our results showed significant increment in their expression indicating the odontoblastic differentiation of DPSCs under various conditions (Figure 1K; p< 0.05 and p < 0.01).

### 3.2 Odontoblast-like differentiating hDPSCs gene profile modulated after TNFα treatment

**FIGURE 2:**
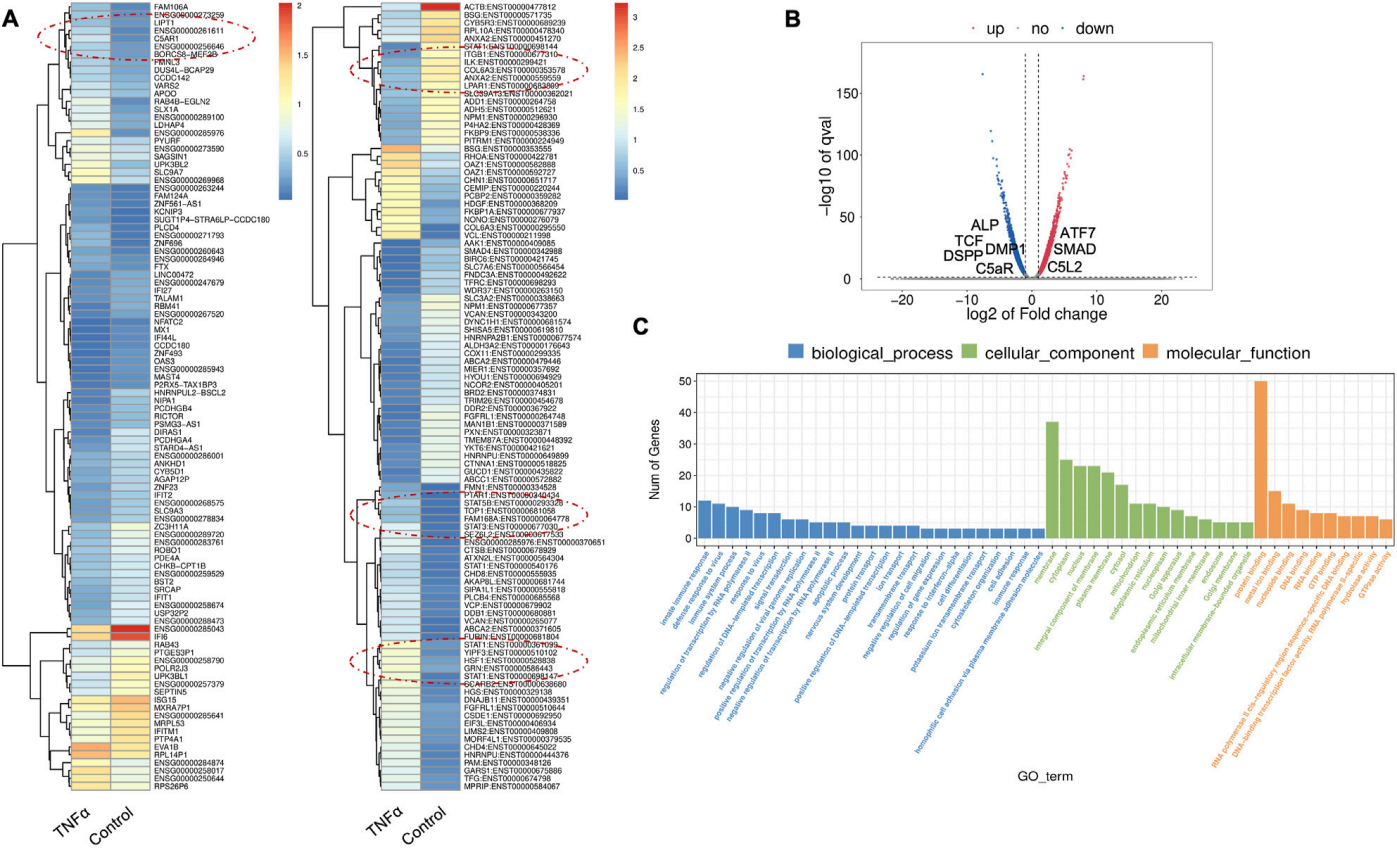
Analysis of differentially expressed genes (DEGs) in RNA-Seq. data. in TNFα-treated odontoblast-like differentiating DPSCs. **(A)** Heatmap of the differentially expressed genes between untreated or control cells and TNFα hDPSCs in dentinogenic media. Red and yellow stripes in the figure represent high-expression genes, while blue stripes represent low-expression genes. Particular genes and transcription factors of interest that are involved in odontoblastic differentiation are red circled from the clusters and shown in the following figure (Figure 4) in the graphs. **(B)** Volcano map of differentially expressed genes (DEGs) between control cells and TNFα hDPSCs in dentinogenic media. The x-axis is the log2 scale of the fold change of gene expression in hDPSCs (log2 (fold change)). Negative values indicate downregulation; positive values indicate upregulation. The y-axis is the minus log10 scale of the adjusted p values (–log10), indicating the significant expression difference level. The blue dots represent significantly upregulated genes with at least twofold change, while the red dots represent significantly downregulated genes with at least twofold change. Key genes have been mentioned along the volcano dots. **(C)** Based on their functions, significantly enriched Gene Ontology (GO) terms were found among control and TNFα-treated hDPSCs. The top GO terms in the enrichment analysis are biological process, cellular component, and molecular function (MF) terms in the enrichment analysis.

TNFα is one of the several pro-inflammatory cytokines involved in tissue regeneration and differentiation (Osta et al., 2014). We evaluated the transcriptional changes in DPSCs during odontoblastic differentiation under the effects of TNFα. Differential gene expression characterized by a heat map (Figure 2A) shows significant differences in upregulation of transcriptional and gene expression verified by a volcano graph of differentially expressed genes (DEGs) distribution (Figure 2B). Biological processes such as immune system regulation, intracellular signal transduction, and cell differentiation were also significantly enriched, indicating a broad impact on cellular functions (Figure 2C).

Major Gene Ontology (GO) assignments among up-regulated and downregulated genes include innate immune response and defense response under Biological Process (BP), an integral component of membrane and cytoplasm under Cellular Component (CC), and protein folding and metal ion binding under Molecular Function (MF) (Figure 2C). Notably, there was a significant increase in the expression of genes such as Signal transducer and activator of transcription (STAT), Collagen VI (COL6), T-cell factor (TCF), Alkaline Phosphatase (ALP), Dentin Sialophosphoprotein (DSPP), and Dentin matrix acidic phosphoprotein 1 (DMP1). STAT is recognized for its role in regulating inflammation and tissue repair, indicating its potential involvement in reparative dentinogenesis. TCF may influence gene expression associated with odontoblast differentiation and the formation of reparative dentin, where its activation could enhance the regenerative capacity of dental tissues following injury. However, dysregulation of TCF could impair proper dentin formation, leading to developmental defects or compromised repair processes. COL6 is crucial for maintaining the structural integrity of the pulp-dentin complex and supporting odontoblast differentiation and function. Its interactions with other extracellular matrix (ECM) components are likely critical for the mineralization and organization of dentin, contributing to the tooth’s mechanical strength and resilience. ALP is an early marker of odontogenic differentiation in pulp cells, playing a vital role in tissue mineralization and calcification, thus essential for repair and regeneration processes (Golub and Boesze-Battaglia, 2007). DSPP and DMP1 work in concert to regulate the formation of reparative dentin; DSPP primarily drives the mineralization process, ensuring the new dentin is sufficiently hard and resilient DSPP (Gibson et al., 2013), while DMP1 plays a key role in organizing the dentin matrix and regulating mineral deposition, ensuring that reparative dentin forms correctly and integrates effectively with existing tooth structure. The upregulation of both DSPP and DMP1 during reparative dentinogenesis underscores their significance in the body’s response to dental injury (Guo et al., 2014). These findings underscore the significant increase in expression of key genes involved in inflammation and regeneration upon TNFα stimulation.

**FIGURE 3:**
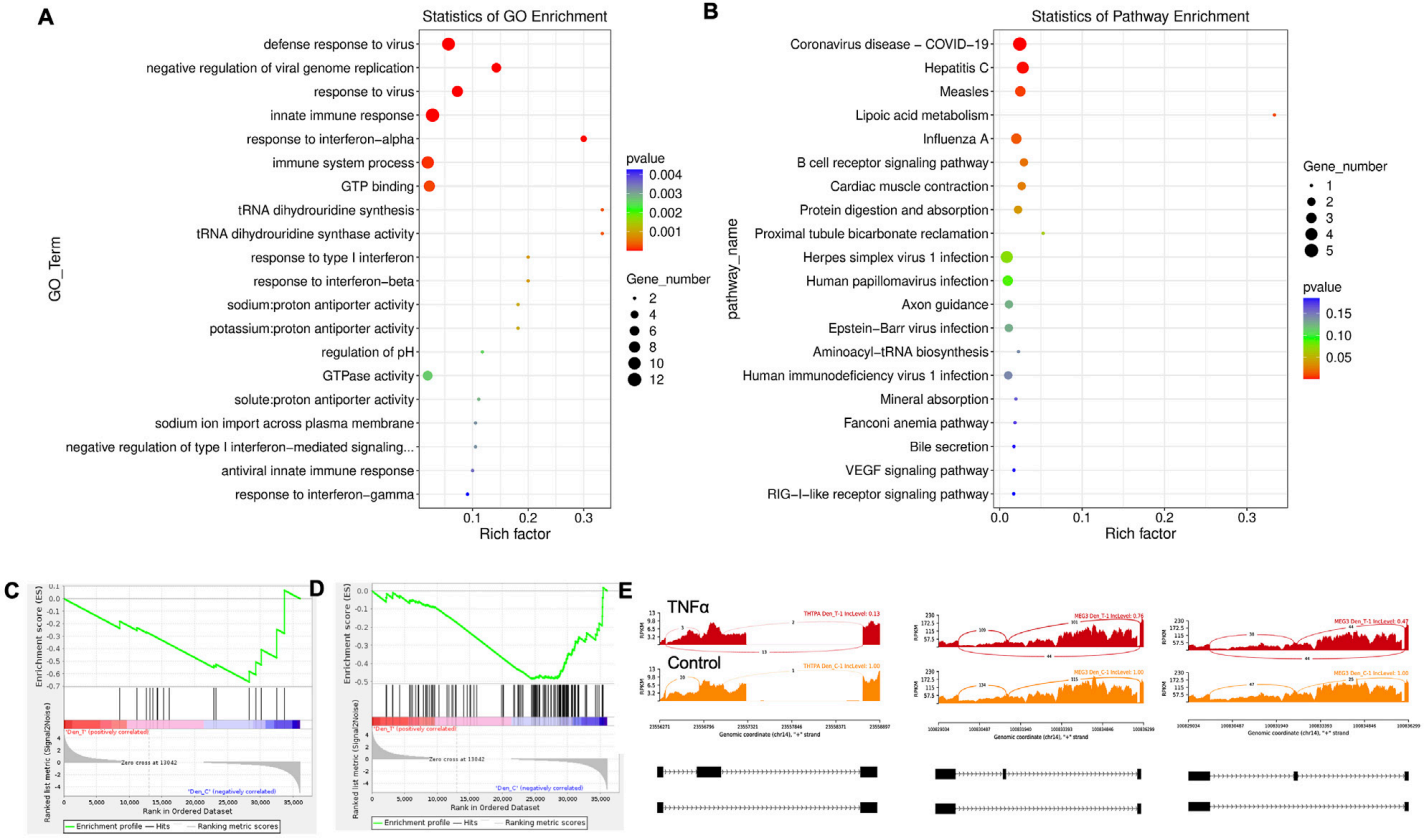
Significantly enriched GO terms can be found in TNFα-treated hDPSCs in dentinogenic media based on their functions compared with the control. **(A,B)** Go-term and Reactome enrichment pathway analysis of up- and downregulated DEGs. The dot plot shows the top enriched Reactome pathways. The size of the dot is based on the gene count enriched in the pathway, and the color of the dot shows the significance of pathway enrichment. **(C,D)** Gene set enrichment analysis was performed on DEGs among control and TNFα-treated cells in dentinogenic media and found various upregulated and downregulated genes against inflammatory response **(C)**, extracellular matrix structural constituents **(D)**. **(E)** Sashimi plots for quantitative visualization of RNA sequencing read alignments. The arched lines indicate splice junctions; the number of reads using that splice junction is indicated on the arched line. Data were examined on sashimi plots, which revealed the number of variants and genomic mutations on chr14 in TNFα-treated cells in dentinogenic media against the control. Red sashimi plots show variants in the TNFα-treated group, and orange shows in the control. While lower black annotations are Read alignments of alternative isoforms and genomic regions of interest.

Twenty significant gene ontology (GO) enrichment groups were identified, including several enriched pathways related to the regulation of immune response (Figures 3A,B), inflammatory response (Figure 3C), and extracellular structural constituents (Figure 3D). The Sashimi plot illustrates RNA-seq read densities across exons and splice junctions, aligning them with the gene’s isoform structure and can be used to quickly screen differentially spliced exons along genomic regions of interest. Figure 3E presents Sashimi plots for three events on chr14 in TNFα-treated cells in dentinogenic media against the control. For the first gene, there were 21 junction reads in the control samples with an inclusion level of 1.00. However, in cells exposed to TNFα, the junction reads decreased to 5, with an inclusion level of 0.13. This suggests that TNFα exposure induces exon skipping in this particular gene. Similarly, gene 2 and gene 3 exhibited 0.76 and 0.47 inclusion levels, respectively, following TNFα treatment, whereas the control had a high inclusion level of 1.00 for both genes. The difference in inclusion levels between TNFα and control suggests that treatment with TNFα results in a decrease in exon inclusion, leading to exon skipping or the production of alternative transcript isoforms. Thus, the results reveal that TNFα treatment significantly impacts the splicing mechanisms of the genes presented, potentially altering their function or expression profiles.

**FIGURE 4:**
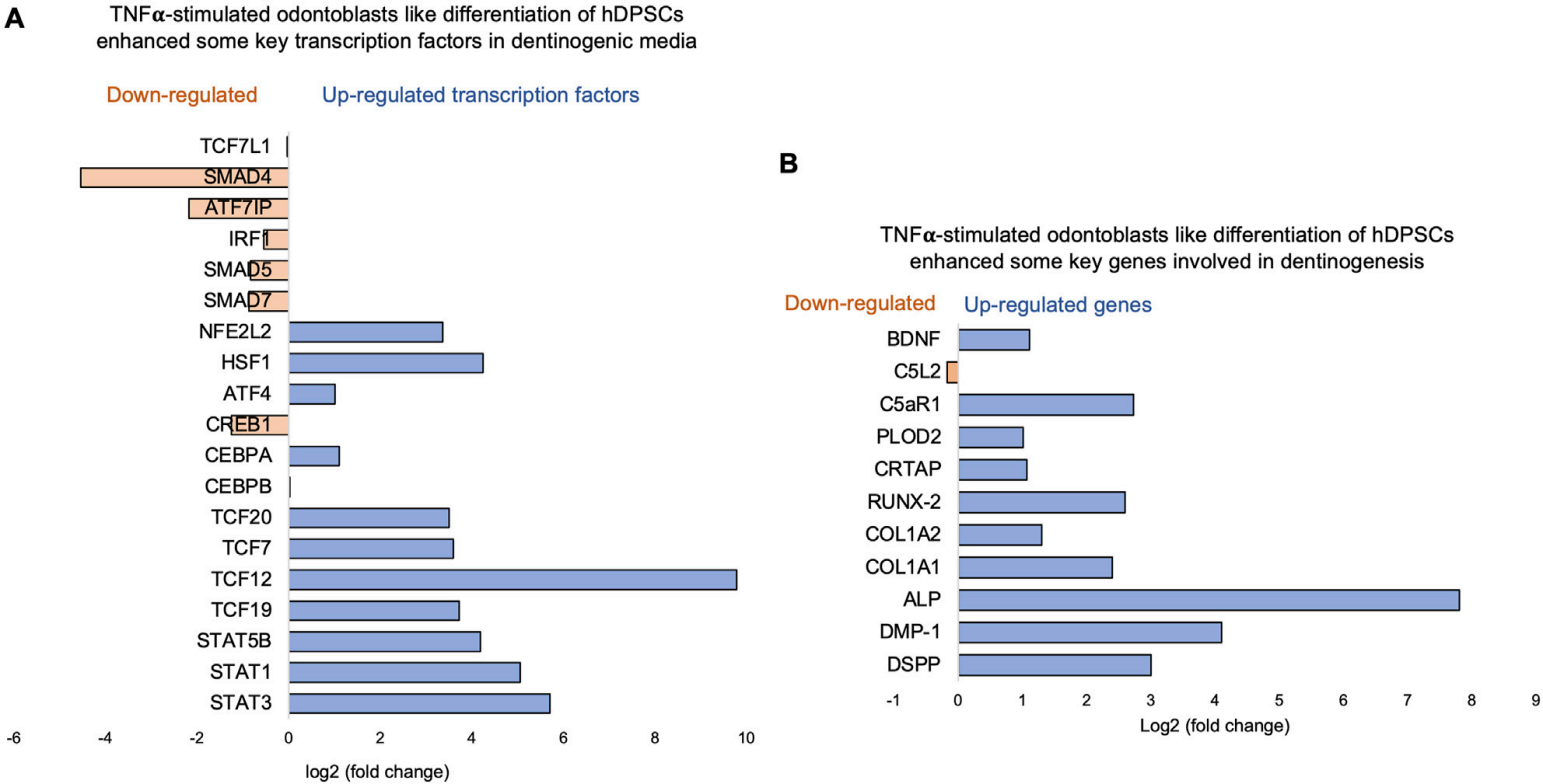
Signaling pathways affected by TNFα in odontoblast-like differentiation of hDPSCs in dentinogenic media. hDPSCs were cultured and treated with or without TNFα for 7 days (twice a week with 3-day intervals). Cell lysates were collected, and RNA was prepared using the RNeasy mini kit (Qiagen), and next-generation RNA sequencing was done using the poly-A-RNA sequencing technique. **(A)** Histogram showing upregulated and activated transcription factors (blue) and repressed or downregulated transcription factors (orange). Notably, TCF (Pezelj-Ribaric et al., 2002; Irfan and Chung, 2023; Tsutsui, 2020; Tsutsui, 2020; Pagella et al., 2020), especially TCF12, is highly upregulated in TNFα-stimulated odontoblasts like differentiated DPSCs. **(B)** Histogram showing upregulated and activated upregulated genes (blue) and repressed or downregulated genes (orange). Notably, key genes involved in dentinogenesis are significantly upregulated in TNFα-stimulated odontoblast-like differentiated DPSCs.

Key transcriptional factors such as lymphoid enhancer factor/T-cell factor (LEF/TCFs) significantly increased (Figure 4A), which work through βthe -catenin pathway to increase BDNF (Zhang et al., 2018), and we have recently reported that enhanced BDNF secretion stimulates DPSCs-mediated dentinogenesis (Kim et al., 2023). TNFα stimulation decreases SMAD activation by inducing NF-κB, suppressing BMP-2 and TGFβ-mediated SMAD signaling. This suppression disrupts osteoblast differentiation and mineralization, leading to impaired bone formation. The implication is that targeting TNFα or NF-κB could preserve SMAD signaling, potentially enhancing bone mass and offering therapeutic strategies for bone-related disorders like osteoporosis (Li et al., 2007). ATF7ip Inhibits Osteoblast Differentiation via Negative Regulation of the Sp7 transcription factor (ref). Also, it is identified as a crucial regulator of CD8^+^ T cell immune responses, influencing their effector and memory functions. Mice with a T cell-specific deletion of ATF7ip show increased expression of Il7r and Il2, leading to enhanced CD8^+^ T cell responses (Sin et al., 2022).

Similarly, key genes known as odontoblast-like differentiation markers, also involved in dentin mineralization, such as ALP, DMP-1, RUNX-2, and DSPP (Chen et al., 2022) were significantly increased (Figure 4B). The results suggest that TNFα treatment significantly modulates the gene expression profile of odontoblast-like differentiating hDPSCs, particularly impacting immune system regulation, intracellular signaling, and cell differentiation processes. The findings indicate that TNFα′s influence on key transcriptional factors and signaling pathways may disrupt osteoblast differentiation and dentinogenesis, highlighting potential therapeutic targets for enhancing bone formation and treating bone-related disorders.

### 3.3 C5L2 silencing enhances immune-regulating and dentinogenic factors

**FIGURE 5:**
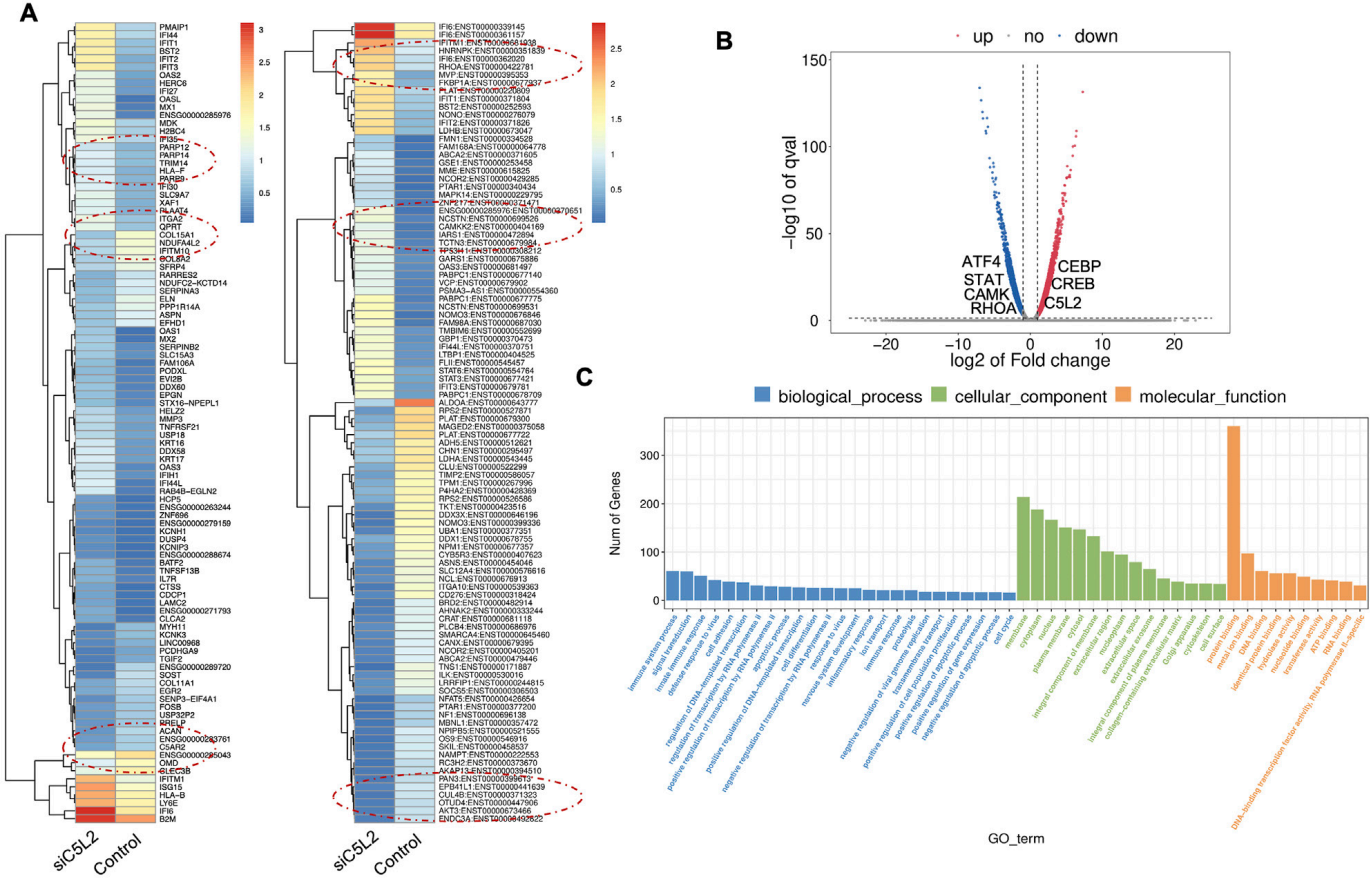
Analysis of differentially expressed genes (DEGs) in RNA-Seq. data in siC5L2-treated odontoblast-like differentiating DPSCs. **(A)** Heatmap of the differentially expressed genes between untreated or control cells and siC5L2-treated hDPSCs in dentinogenic media. Red and yellow stripes in the figure represent high-expression genes, while blue stripes represent low-expression genes. Particular genes and transcription factors of interest that are involved in odontoblastic differentiation are red circled from the clusters and shown in the following figure (Figure 7) in the graphs. **(B)** Volcano map of differentially expressed genes (DEGs) between control cells and siC5L2 hDPSCs in dentinogenic media. The x-axis is the log2 scale of the fold change of gene expression in hDPSCs (log2 (fold change)). Negative values indicate downregulation; positive values indicate upregulation. The y-axis is the minus log10 scale of the adjusted p values (–log10), indicating the significant expression difference level. The blue dots represent significantly upregulated genes with at least twofold change, while the red dots represent significantly downregulated genes with at least twofold change. Key genes have been mentioned along the volcano dots. **(C)** Their functions are significantly enriched in Gene Ontology (GO) terms among control and siC5L2-treated hDPSCs.

Recent studies, including ours, have identified the activation of the complement system—a key early response to tissue damage—as an additional source of regenerative signals promoting dentin regeneration following carious injuries. We have reported that C5L2 silencing and KO enhance dentinogenesis by regulating BDNF via the p38 pathway (Irfan and Chung, 2023; Irfan et al., 2024). Here, we examined the differential gene expression in siC5L2-treated odontoblastic-like differentiating DPSCs. The heat map shows changes in gene expression (Figure 5A), and the volcano graph of DEGs shows upregulated genes against -log10 plot (Figure 5B), while biological processes such as immune system regulation, signal transduction, and inflammatory response were also significantly enriched, indicating a broad impact on cellular functions (Figure 5C).

The major GO terms identified among the genes include innate immune response and signal transduction under Biological Process (BP); integral component of membrane and cytoplasm under Cellular Component (CC); and protein folding and metal ion binding under Molecular Function (MF) (Figure 5C). Among these, there was a notable increase in the expression of genes such as Signal Transducer and Activator of Transcription 3 (STAT3), Signal Transducer and Activator of Transcription 6 (STAT6), and Ras homolog family member A (RhoA). STAT3, typically activated in response to inflammation and tissue injury, promotes the survival and proliferation of hDPSCs and odontoblasts. It also facilitates the differentiation of DPSCs into odontoblast-like cells, ensuring that an adequate number of DPSCs are available for the reparative process (Chan et al., 2023). STAT6 regulates genes involved in extracellular matrix production and remodeling, which are critical for maintaining and repairing dentin. RhoA plays a key role in regulating the cytoskeletal structure of odontoblasts, influencing their migration and positioning during dentinogenesis. Additionally, RhoA signaling is crucial for differentiating DPSCs into odontoblast-like cells and impacts the secretion and organization of the dentin matrix. RhoA also helps odontoblasts adjust their cytoskeleton and function in response to mechanical stress, thereby maintaining the integrity of the dentin-pulp complex (Xue et al., 2013).

**FIGURE 6:**
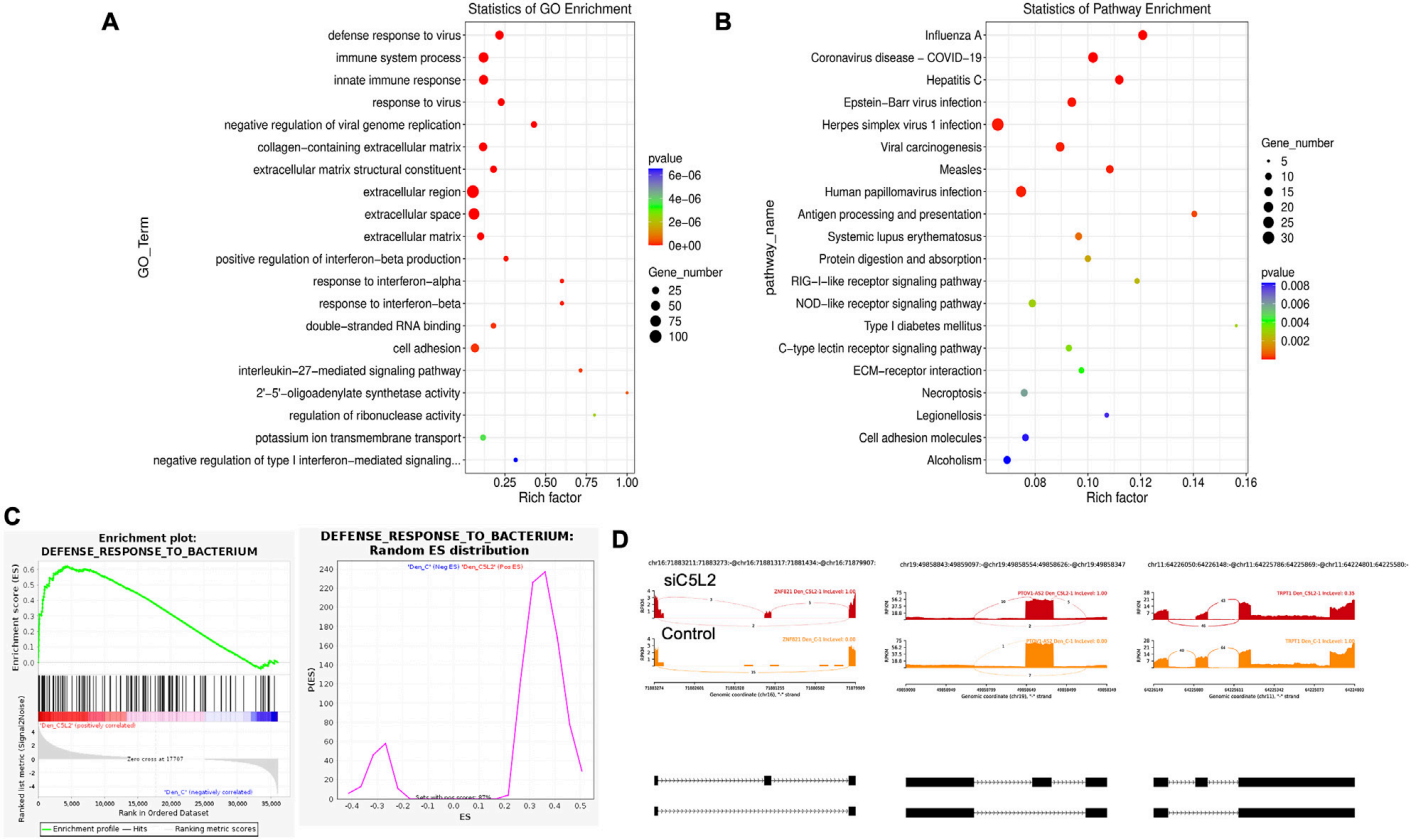
The top GO terms in the enrichment analysis among biological process, cellular component, and molecular function (MF) terms in the enrichment analysis in siC5L2-treated odontoblast-like differentiating DPSCs. Significantly enriched GO terms can be found in TNFα-treated hDPSCs in dentinogenic media based on their functions compared with the control. **(A,B)** Go-term and Reactome enrichment pathway analysis of up- and downregulated DEGs. The dot plot shows the top enriched Reactome pathways. The dot size is based on the gene count enriched in the pathway, and the dot color shows the significance of pathway enrichment. **(C)** Gene set enrichment analysis was performed on DEGs among control and siC5L2-treated cells in dentinogenic media, and various upregulated and downregulated genes were found to be against bacterial response. Enrichment plot and Random ES distribution show that the defense response against bacteria has been enhanced after C5L2 silencing. **(D)** Sashimi plots for quantitative visualization of RNA sequencing read alignments. The arched lines indicate splice junctions; the number of reads using that splice junction is indicated on the arched line. Data were examined on sashimi plots, revealing variants and genomic mutations on chr16, chr19, and chr11 in siC5L2-treated cells in dentinogenic media against the control. Red sashimi plots show variants in the siC5L2-treated group, and orange shows in the control. While lower black annotations are Read alignments of alternative isoforms and genomic regions of interest.

Twenty significant GO enrichment groups were identified, including several enriched pathways related to the regulation of immune response, collagen-containing extracellular matrix, and cell adhesion (Figures 6A,B), which are considered critical in the regeneration process (Julier et al., 2017). In caries, the innate immune response against bacteria is triggered (Julier et al., 2017; Nishita et al., 2000), which is important in regulating oral health. In our results, the Enrichment plot and Random ES distribution show that the defense response against bacteria has been significantly enhanced after C5L2 silencing (Figure 6C). Sashimi plots for quantitative visualization of RNA sequencing read alignments. Data were examined on sashimi plots, which revealed the number of variants and genomic mutation on chr16, chr19, and chr11 in siC5L2 treated cells in dentinogenic media against control (Figure 6D). For LOC, inclusion level (Inclevel) is reported as 1.00, indicating that this splicing or the exon skipping is fully favored in siC5L2. Similarly, gene 2 shows 1 inclusion level following gene silencing, whereas siC5L2 treatment leads to 0.35 inclusion levels in gene 3 against the control. This comparison demonstrates that siC5L2 treatment favors exon skipping in two of the three genes shown, leading to a different transcript isoform than the control.

### 3.4 Transcription factor 7-like 2 increased by C5L2 silencing

**FIGURE 7:**
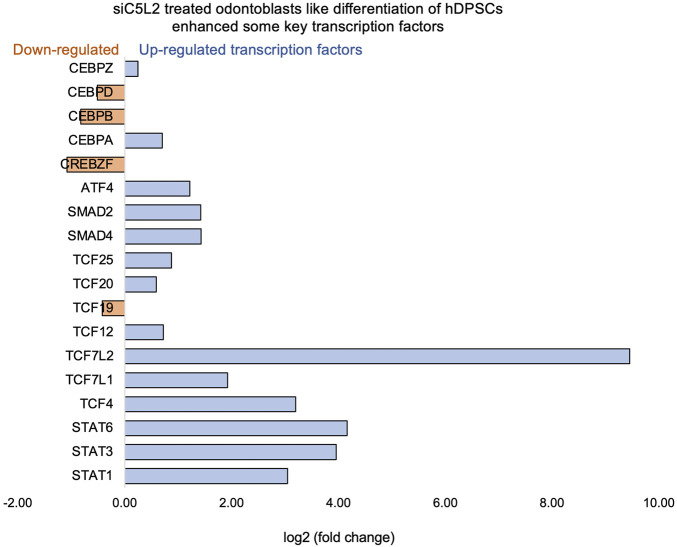
Signaling pathways affected by siC5L2 in odontoblast-like differentiation of hDPSCs in dentinogenic media. hDPSCs were cultured and treated with or without siC5L2 and differentiated for 7 days. Cell lysates were collected, and RNA was prepared using the RNeasy mini kit (Qiagen), and next-generation RNA sequencing was done using the poly-A-RNA sequencing technique. Histogram showing upregulated and activated transcription factors (blue) and repressed or downregulated transcription factors (orange). Notably, TCF (Cooke, 2019; Pezelj-Ribaric et al., 2002; Irfan and Chung, 2023; Pagella et al., 2020; Pagella et al., 2020; Martin, 2011), especially TCF7L2, is highly upregulated in siC5L2-stimulated odontoblast-like differentiated DPSCs.

β-catenin and LEF/TCF form a complex with SMAD4 (Nishita et al., 2000), and Silverio et al. (Silvério et al., 2012) proposed that β-catenin and SMAD proteins are needed to activate cementoblast/osteoblast gene expression and promote full differentiation. Bem et al. (Bem et al., 2019) proposed that alterations in the activity of LEF/TCFs, and particularly of transcription factor 7-like 2 (TCF7L2), result in defects previously associated with neuropsychiatric disorders, including imbalances in neurogenesis and oligodendrogenesis. The canonical Wnt signaling pathway plays an important role in tooth development by pulp capping, enhancing cell proliferation, and inducing odontoblast differentiation (Hunter et al., 2015; Hara et al., 2021). Accumulated β-catenin translocates into the nucleus and binds to the transcription factor TCF/LEF to promote transcription (van Amerongen and Nusse, 2009). Again, our results showed that siC5L2 treatment enhanced BDNF secretion-related transcriptional factors, ultimately contributing to dentinogenesis, including TCF/LEF (especially TCF7L2) and SMADs (Figure 7).

The signal transducer and activator of transcription (STAT) protein family is an intracellular transcription factors that mediate many aspects of cellular immunity, proliferation, apoptosis, and differentiation. These transcription factors, activated through the JAK-STAT pathway, play a crucial role in stem cell proliferation by promoting the transcription of genes associated with cell growth (Pourrajab et al., 2013). The results suggest that silencing C5L2 increases the expression of transcription factor TCF7L2, which plays a critical role in odontoblast differentiation and dentinogenesis through the Wnt/β-catenin and SMAD signaling pathways. This indicates that targeting C5L2 could enhance these pathways, potentially improving tooth regeneration and repair processes.

### 3.5 Combined effects of TNFα and siC5L2 on immune regulators and dentinogenic markers

**FIGURE 8:**
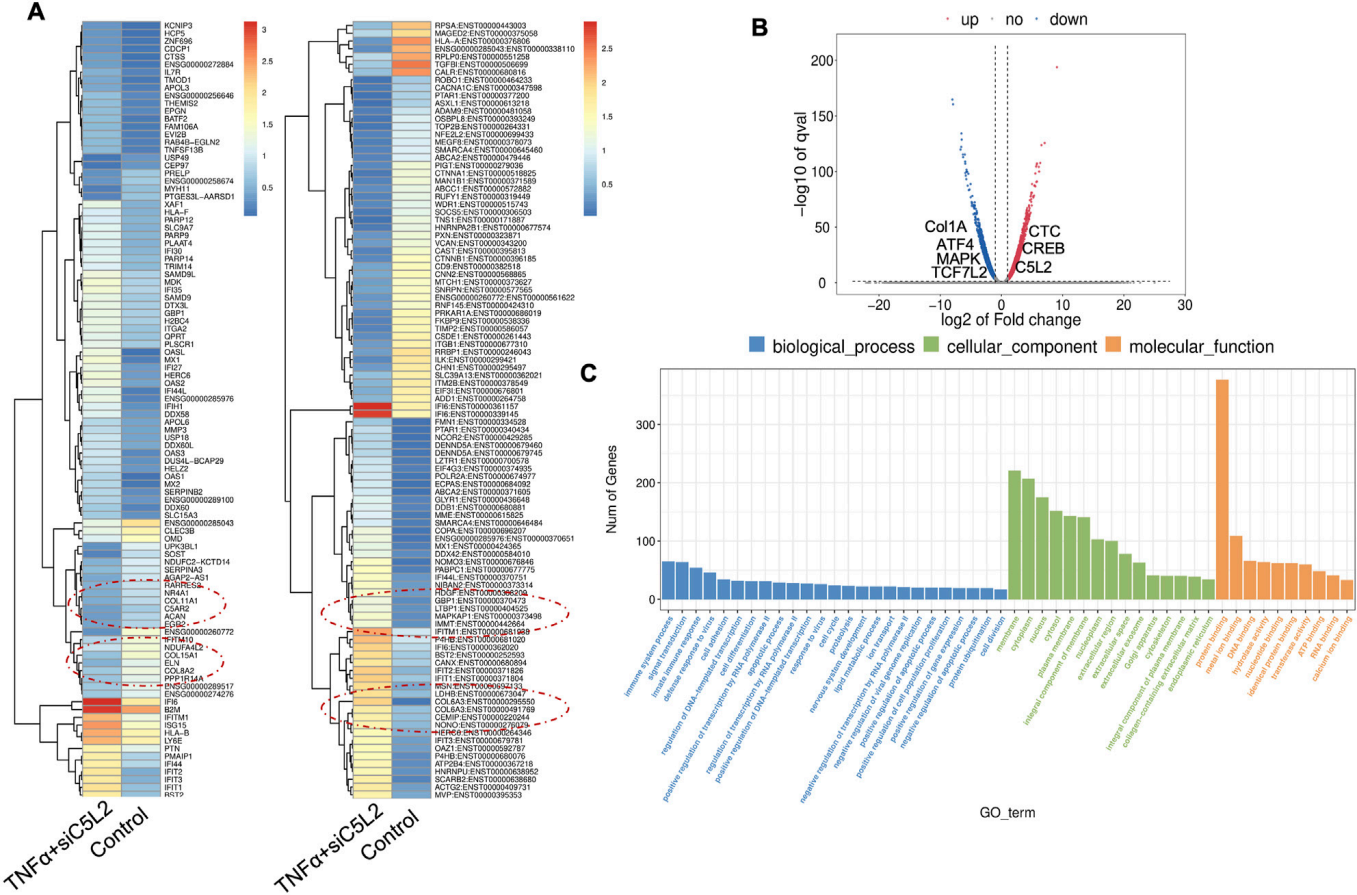
Analysis of differentially expressed genes (DEGs) in RNA-Seq. data. in TNFα and siC5L2 combined groups. **(A)** Heatmap of the differentially expressed genes between untreated or control cells and TNFα-treated C5L2-silenced hDPSCs in dentinogenic media. Red and yellow stripes in the figure represent high-expression genes, while blue stripes represent low-expression genes. Particular genes and transcription factors of interest that are involved in odontoblastic differentiation are red circled from the clusters and shown in the following figure (Figure 10) in the graphs. **(B)** Volcano map of differentially expressed genes (DEGs) between control cells and siC5L2 hDPSCs in dentinogenic media. The x-axis is the log2 scale of the fold change of gene expression in hDPSCs (log2 (fold change)). Negative values indicate downregulation; positive values indicate upregulation. The y-axis is the minus log10 scale of the adjusted p values (–log10), indicating the significant expression difference level. The blue dots represent significantly upregulated genes with at least twofold change, while the red dots represent significantly downregulated genes with at least twofold change. Key genes have been mentioned along the volcano dots. **(C)** Based on their functions, significantly enriched Gene Ontology (GO) terms were found among control and TNFα+siC5L2-treated hDPSCs.

Recently, we have reported the synergistic effects of TNFα and siC5L2 treatment on BDNF secretion and dentinogenesis (Irfan and Chung, 2023; Irfan et al., 2024; Kim et al., 2023). Here, we have examined their effects on differential gene profiling of TNFα and siC5L2-treated odontoblastic-like differentiating DPSCs. The heat map shows differential gene expression in both groups (Figure 8A), while the volcano graph of DEGs shows upregulated genes against -log10 plot (Figure 8B), and biological processes such as innate immune response, signal transduction, cell differentiation, and proliferation were significantly enriched, indicating a broad impact on cellular functions (Figure 8C).

The primary GO terms identified among the genes include innate immune response and signal transduction under Biological Process (BP); integral component of membrane and cytoplasm under Cellular Component (CC); and protein folding and metal ion binding under Molecular Function (MF) (Figure 8C). COL6A and Interferon alpha-inducible protein 6 (IFI6). COL6A plays a crucial role in reparative dentinogenesis by contributing to the formation and organization of the extracellular matrix, supporting odontoblast differentiation, and enhancing the tissue’s ability to respond to injury and mechanical stress (Baldión et al., 2018). Its involvement in these processes makes it an important factor in maintaining the health and integrity of dental tissues, particularly following injury. IFI6 modulates the innate immune response by interacting with components of the retinoic acid-inducible gene I (RIG-I) signaling pathway, maintaining a balance in the immune response, preventing overactivation that could lead to excessive inflammation or tissue damage (Villamayor et al., 2023).

**FIGURE 9:**
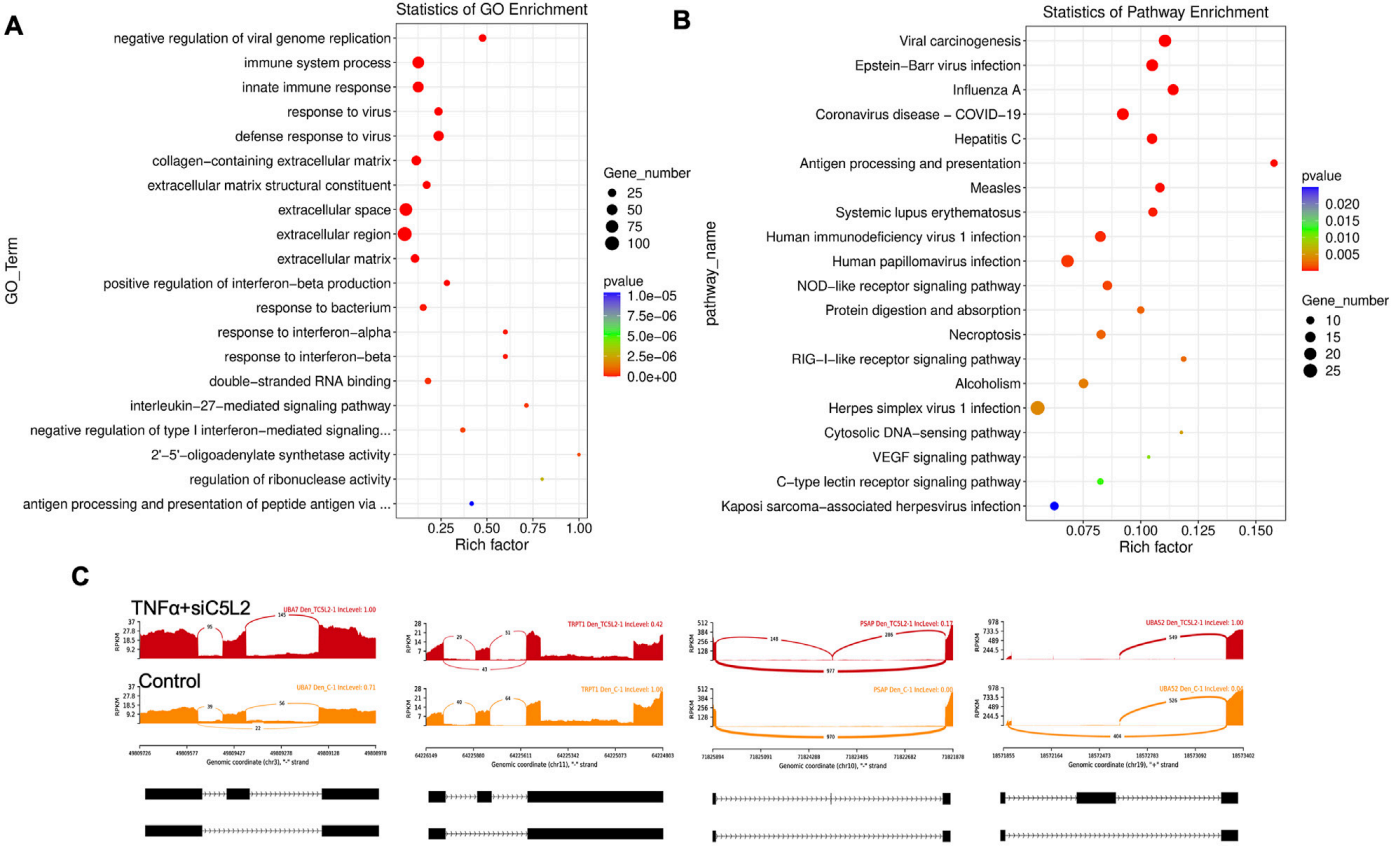
The top GO terms in the enrichment analysis are biological process, cellular component, and molecular function (MF) terms in the enrichment analysis in RNA-Seq. data. in TNFα and siC5L2 combined groups. Significantly enriched GO terms can be found in TNFα-treated C5L2-silenced hDPSCs in dentinogenic media based on their functions compared with the control. **(A,B)** Go-term and Reactome enrichment pathway analysis of up- and downregulated DEGs. The dot plot shows the top enriched Reactome pathways. The size of the dot is based on the gene count enriched in the pathway, and the color of the dot shows the significance of pathway enrichment. **(C)** Sashimi plots for quantitative visualization of RNA sequencing read alignments. The arched lines indicate splice junctions; the number of reads using that splice junction is indicated on the arched line. Data were examined on sashimi plots, which revealed the number of variants and genomic mutations on chr3, chr11, Chr10, and chr19 in TNFα-treated C5L2-silenced DPSCs in dentinogenic media against the control. Red sashimi plots show variants in the TNFα+siC5L2-treated group, and orange shows in the control. While lower black annotations are Read alignments of alternative isoforms and genomic regions of interest.

Similarly, as mentioned above, twenty significant GO enrichment groups were identified, including several enriched pathways related to the regulation of immune response, collagen-containing extracellular matrix and structural constituent, and response to the bacterium (Figures 9A,B) that contribute to the regeneration process (Julier et al., 2017). Sashimi plots for quantitative visualization of RNA sequencing read alignments revealed the number of variants and genomic mutations on chr3, chr11, Chr10, and chr19 in TNFα-treated C5L2-silenced DPSCs in dentinogenic media against control (Figure 9C).

**FIGURE 10:**
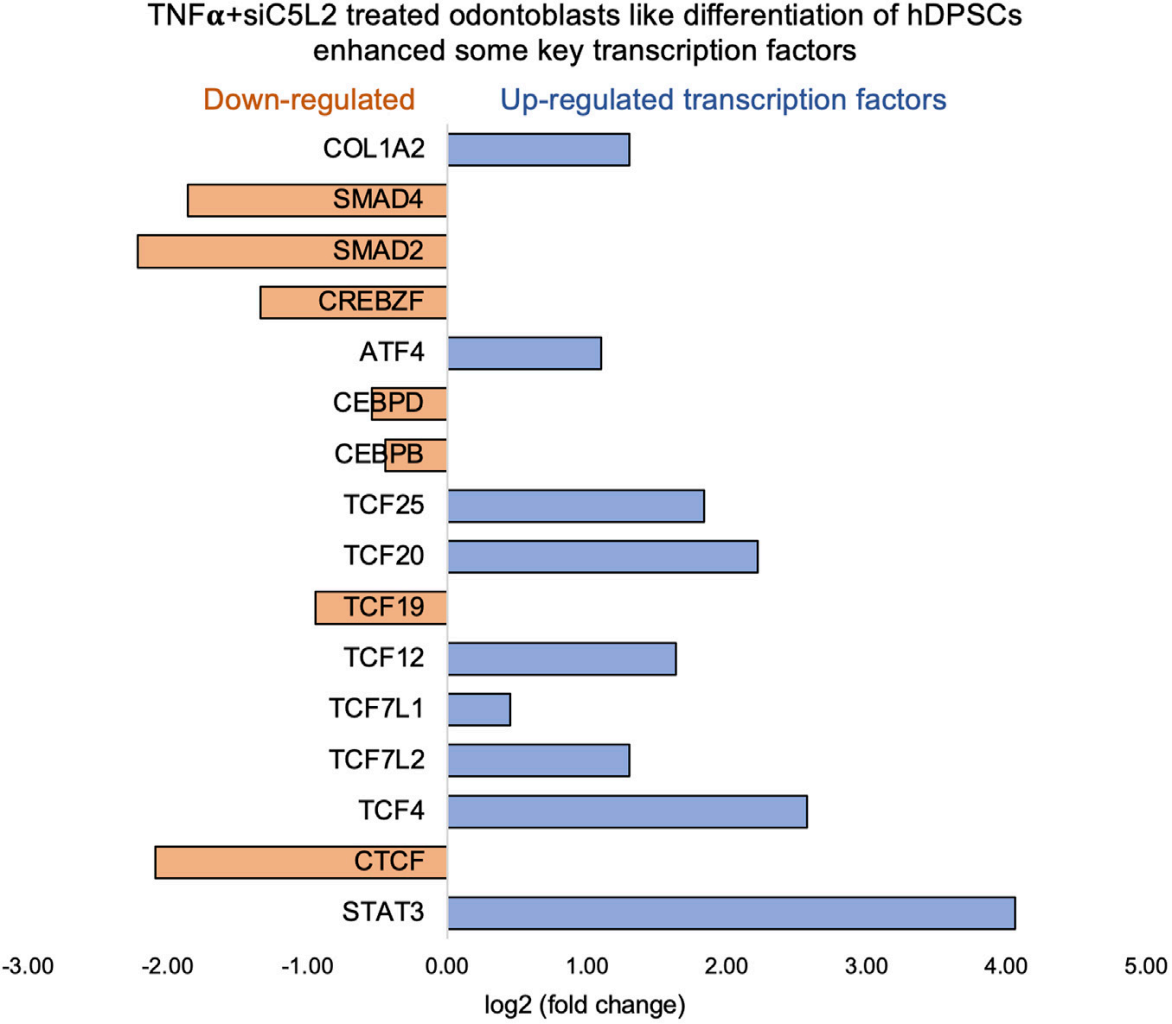
Signaling pathways affected by C5L2 silencing in odontoblast-like differentiation of hDPSCs in dentinogenic media stimulated by TNFα. hDPSCs were cultured and treated with or without TNFα (twice a week) in C5L2-silenced DPSCs and differentiated for 7 days. Cell lysates were collected, and RNA was prepared using the RNeasy mini kit (Qiagen), and next-generation RNA sequencing was done using the poly-A-RNA sequencing technique. Histogram showing upregulated and activated transcription factors (blue) and repressed or downregulated transcription factors (orange). Notably, TCF (Cooke, 2019; Irfan and Chung, 2023; Pagella et al., 2020; Pagella et al., 2020; Martin, 2011), especially TCF4, is highly upregulated in C5L2-silenced TNFα-stimulated odontoblast-like differentiated DPSCs.

The Chr3 showed a strong splice junction connecting two exons with a high inclusion level of 1.00 in TNFα-treated C5L2-silenced DPSCs; however, the control also had a splice junction, but it connected a different set of exons, with an inclusion level of 0.71. At the locus on chromosome 10, there is a large loop indicating an exon-skipping event, with an inclusion level of 0.17 in TNFα-treated C5L2-silenced DPSC in dentinogenic media. At the same time, the control groups show an inclusion level of 0.00, indicating that this exon is completely skipped. For the locus on chromosome 19, control cells have a very low inclusion level (0.04), suggesting that this exon is almost entirely skipped. In contrast, TNFα-treated C5L2-silenced DPSCs with a high inclusion level of 1.00, indicating that this exon is fully included in the transcripts. Overall, these results highlight how TNFα treatment combined with siC5L2 treatment significantly alters the splicing patterns of the genes shown, leading to different transcript isoforms compared to the control cells. Similarly, TCF/LEF transcription factor expressions increased (Figure 10).

### 3.6 Real-time PCR confirms the expression of TCF family transcription factors and the enhancement of dentinogenic markers

**FIGURE 11:**
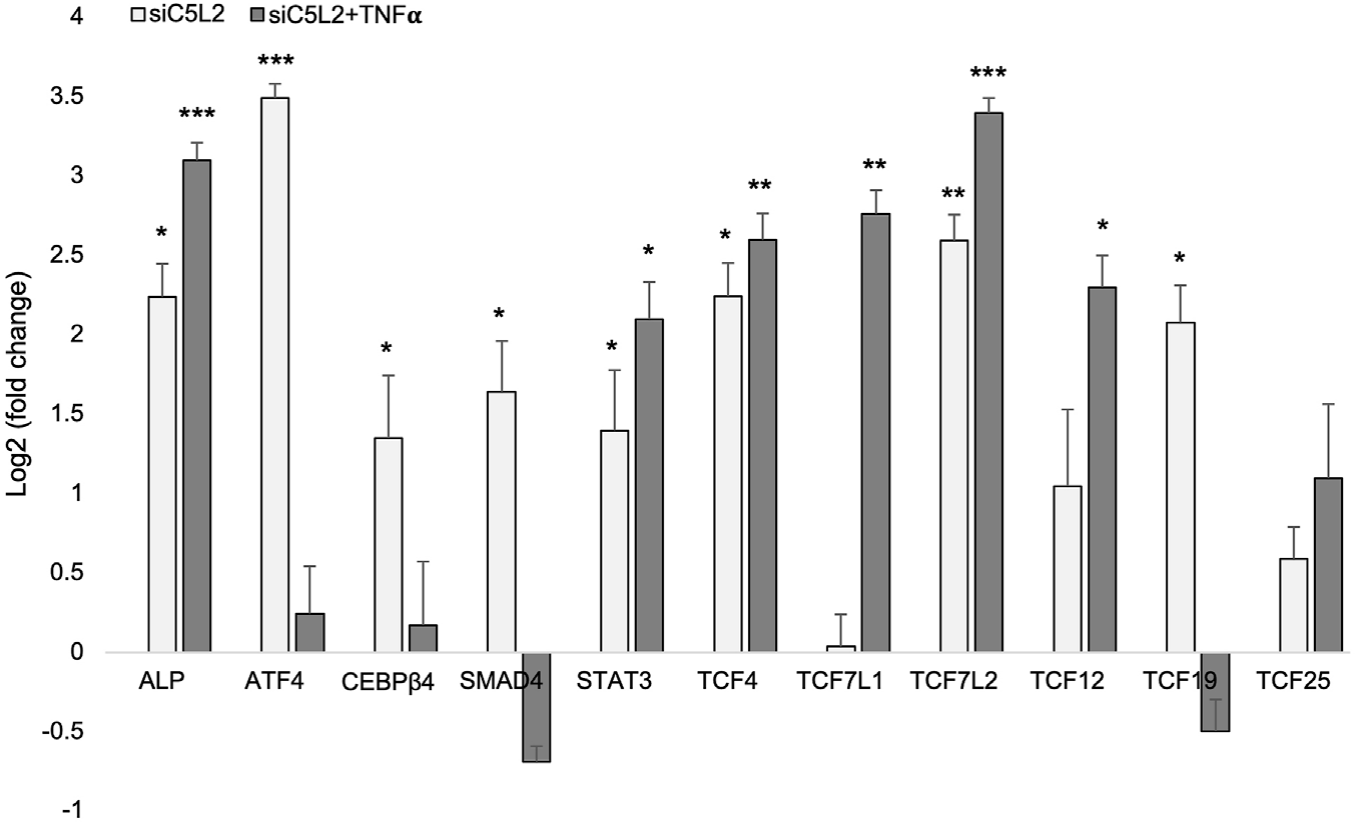
mRNA expression of TCF family transcription factors and other genes. mRNA expression during the odontogenic differentiation was quantified by real-time PCR. The elevated level of these markers represents odontoblast-like differentiation of DPSCs. *p < 0.05, **p < 0.01 and ***p < 0.001 vs. control fold change. The bar graph shows the mean ± SD of at least three independent experiments (n = 3) in duplicate.

We further evaluated the expression of various transcription factors and genes from sequencing by real-time PCR, and our results confirmed the expression of the TCF family transcription factors and other genes involved in dentinogenesis (Figure 11).

## 4 Discussion

It is widely recognized that the regeneration of the dental-pulp complex is closely tied to inflammation. In caries, bacteria and their toxins affect odontoblasts (Shivakumar et al., 2009), while the extension of bacterial infection to dentin affects the behavior of DPSCs in regeneration (Farges et al., 2015). Understanding the impact of inflammatory responses on DPSCs is crucial for uncovering dentin and pulp regeneration mechanisms. Due to the limited data available, this study takes a comprehensive approach to reveal this complex process and to identify key downstream molecules. Our data indicate that DPSCs show significant changes in distinct gene expression profiles and transcriptomic alterations during odontoblastic differentiation after inflammatory stimulation. These include DPSS (3-fold increase), DMP-1 (4.1-fold increase), and alkaline phosphatase (ALP, 7.8-fold increase), indicating the critical role of inflammation in dentinogenesis.

Inflammation and immune responses are modulated by numerous pro-inflammatory cytokines, including TNF,α which acts through various pathways. The pro-inflammatory effects of this cytokine are well-documented (Biton et al., 2012) and are involved in many osteo-immunological diseases, including rheumatoid arthritis (Feldmann and Maini, 2008). Previous studies have proven the positive effect of low concentrations of TNFα on increased osteogenic differentiation by upregulation of RUNX-2, ALP, and OCN levels (Huang et al., 2011; Glass et al., 2011). This dual effect of TNFα on osteogenic differentiation is directly related to its concentration, cell type, and exposure time (Osta et al., 2014). We had established that inflammation is directly involved in complement C5a-mediated dentin repair in DPSCs (Irfan et al., 2024; Kim et al., 2024) and other cell types like stem cells of dental apical papilla (Wang et al., 2017) and bone marrow-derived mesenchymal stem cells (Kim et al., 2013). In our current study, the low concentration and minimal exposure of TNFα on odontoblast-like differentiating DPSCs for a specific time has increased the expression of osteogenic markers such as RUNX-2, ALP, DMP-1, and DSPP while also increasing the downstream transcriptional factors like LEF/TCF and CEBP-β. β-catenin and LEF/TCF form a complex with SMAD4 (Nishita et al., 2000; Silvério et al., 2012) and are needed to activate osteoblast gene expression and promote full differentiation. LEF/TCF and nuclear β-catenin can also act independently of each other by interacting with other transcription factors. Still, the majority of β-catenin binds to chromatin via LEF/TCFs (Tomas et al., 2014; Schuijers et al., 2014). Among the TCF family, particular attention is given to the role of TCF7L2, for having a central role in neural stem cell differentiation and postmitotic differentiation of some brain regions, impairments of which might contribute to mental disorders (Bem et al., 2019). The specific roles of LEF1, TCF7L2, and TCF7L1 in hippocampal developmental neurogenesis have been investigated in gene knockout studies, suggesting a redundant role for LEF1 and other LEF1/TCF family members. TCF7L1 knockout did not result in any hippocampal alterations, but the size of the hippocampus’s granule and pyramidal cell layers was markedly reduced in TCF7L2 knockout mice (Hara et al., 2021; Chodelkova et al., 2018). In our results, C5L2 silencing showed significantly enhanced expression of TCF7L2, indicating the role of C5L2 silencing in the neural regeneration process and odontoblastic differentiation of DPSCs.

The STAT, SMAD, and ATF transcription factors play pivotal roles in inflammation-induced regeneration, particularly in processes like dentinogenesis and tissue repair. STAT proteins, activated through the JAK-STAT pathway, are critical mediators of cellular immunity, proliferation, apoptosis, and differentiation. In the context of dentinogenesis, STATs promote the transcription of genes essential for stem cell proliferation and differentiation, thereby contributing to the regeneration of dental tissues (Pourrajab et al., 2013). SMAD proteins, which are key mediators of the TGF-β signaling pathway, are crucial for osteoblast and odontoblast differentiation. However, inflammation-induced factors like TNFα can inhibit SMAD signaling by activating NF-κB, impairing differentiation and mineralization. This disruption can adversely affect dentinogenesis and bone formation, making regulating SMAD activity a potential therapeutic target for enhancing dental and bone regeneration (Li et al., 2007). ATF genes, particularly ATF7ip, have been identified as negative regulators of osteoblast differentiation by influencing transcription factors such as Sp7. Additionally, ATF7ip modulates immune responses, which can have significant implications for inflammation-driven regenerative processes. Therefore, targeting these transcription factors could offer new strategies to modulate inflammation, enhance tissue regeneration, and improve outcomes in dental and bone repair therapies (Sin et al., 2022). These pathways collectively underscore the complex interplay between inflammation, transcriptional regulation, and tissue regeneration, highlighting the importance of fine-tuning these molecular signals to promote effective dentinogenesis and regeneration.

The absence of biomarkers in early detection and drug resistance is a principal cause of treatment failure in various disease conditions. RNA-Seq has expanded the knowledge of molecular-level pathology progression and novel tissue transcriptional changes. RNA-Seq technology has emerged as a powerful tool for identifying functional genes and pathways in diagnosing various disease mechanisms (Wang et al., 2019). It has discrete advantages over former methodologies and has transformed our understanding of the complex and dynamic nature of the transcriptome. It provides a more detailed, unbiased, quantitative view of gene expression, alternative splicing, and allele-specific expression. Recent advancements in the RNA-Seq workflow, from sample preparation to sequencing platforms to bioinformatic data analysis, have enabled deep profiling of the transcriptome and the opportunity to explore various physiological and pathological conditions (Wang et al., 2019; Kukurba and Montgomery, 2015). However, available methods had various limitations and restraints: the obligation for prior knowledge of the sequences, problematic cross-hybridization artifacts in the analysis of highly similar sequences, and limited ability to accurately quantify lowly expressed and very highly expressed genes (Kukurba and Montgomery, 2015; Casneuf et al., 2007; Shendure, 2008).

Stem cell therapy is a newly proposed technique for several diseases, and human DPSCs have been under consideration in regenerative medicine for the last decade. DPSCs have a high capacity for differentiation and regeneration due to multipotency. Previous studies have described the differentiation potential of DPSCs, including odontogenic, osteogenic, and neurogenic potential (Xiao and Nasu, 2014; Arthur et al., 2008), and DPSCs have been successfully used in bone tissue engineering (Paino et al., 2017). BM-MSCs are multipotent stem cells characterized by self-renewal and multilineage differentiation. At the same time, DPSCs display MSC-like characteristics but with easy accessibility, noninvasive isolation, limited ethical concerns, and high proliferation capacity. DPSCs are thought to be promising stem cell sources for clinical use (Yamada et al., 2019). Yamada et al. (Yamada et al., 2019) have summarized the regenerative capabilities of DPSCs in various systemic diseases, especially concerning neural regenerative capacity compared to BM-MSCs, and concluded that DPSCs have comparatively much greater therapeutic potential. In our previous studies, we have used human DPSCs to study odontoblast-like differentiation and their effects on dentinogenesis (Irfan et al., 2024; Kim et al., 2023; Irfan et al., 2022b), and found their suitability to study them in osteogenic differentiation studies.

The complement system is an early response to tissue damage and a critical component of innate immunity and inflammation (Ricklin et al., 2010; Chmilewsky et al., 2014). This could be observed during bacterial infection and caries onset, including the recruitment of immune cells due to the production of anaphylatoxin C5a and some opsonins (Cruvinel et al., 2010). Beyond its role in immunity, the complement system participates in tissue regeneration (Ignatius et al., 2011). Another study has explained the linkage between inflammation and dental tissue regeneration through complement activation (Bergmann et al., 2020) and between inflammation and bone regeneration in BM-MSCs (Liu et al., 2018). It is noteworthy that complement receptor C5aR1 was also increased with treatment of TNFα, indicating the complement C5a activation and involvement in the dentinogenic process and osteogenic differentiation of DPSCs, confirming our previous studies on DPSCs-mediated stem cell therapy and regeneration process (Irfan et al., 2022a; Irfan et al., 2022b). On the other hand, C5aR2 (C5L2) was not affected or reduced a little, while the bacterial defense response was activated in the siC5L2-treated group, showing its positive effect in various ailments. Our RNA profiling data indicate C5L2`s critical role in governing the signaling pathway during inflammatory dentinogenesis. Previously, we have been working on the C5L2 receptor and its involvement in inflammation-mediated dentinogenesis (Irfan and Chung, 2023; Irfan et al., 2024). Together with the current study’s outcome, the evidence of indirect involvement of C5L2 in the regulation of dentin reparative process via BDNF/TrkB regulation and DSPP and DMP-1 increment, and upregulation of downstream signaling through a key transcriptional factor, i.e., TCF7L12 and β-catenin, proposes its as a potential target candidate to be considered to explore further in the field of stem cell-based regenerative therapy. As C5L2 is still considered a controversial receptor (also known to work against C5aR1), there’s limited information available (Li et al., 2013). We continue to explore its mechanistic aspects in the field of regenerative medicine.

## 5 Conclusion

Taken together, bulk RNA analysis revealed the key transcription factors and differentially expressed genes involved in inflammation-induced odontoblast-like differentiating DPSCs, indicating a critical role of TNFα and complement C5a receptor 2 silencing. This may direct future studies on specific transcription factors and the role of TNFα-induced inflammation in stem cell-mediated regeneration therapies.

## Data Availability

The datasets presented in this study can be found in online repositories. The names of the repository/repositories and accession number(s) can be found below: https://uofi.app.box.com/folder/302218883136, UofC Box folder.
